# Modeling the Hysteresis Loop of Ultra-High Permeability Amorphous Alloy for Space Applications

**DOI:** 10.3390/ma11112079

**Published:** 2018-10-24

**Authors:** Michał Nowicki, Roman Szewczyk, Tomasz Charubin, Andriy Marusenkov, Anton Nosenko, Vasyl Kyrylchuk

**Affiliations:** 1 Institute of Metrology and Biomedical Engineering, Warsaw University of Technology, 02-495 Warsaw, Poland; szewczyk@mchtr.pw.edu.pl (R.S.); charubin@mchtr.pw.edu.pl (T.C.); 2 Lviv Center of the Institute of Space Research, 79060 Lviv, Ukraine; marand@isr.lviv.ua; 3 V. Kurdyumov Institute for Metal Physics of NAS of Ukraine, 03142 Kyiv, Ukraine; nosenko@imp.kiev.ua (A.N.); Kyrylchuk_Vasyl@nas.gov.ua (V.K.)

**Keywords:** high permeability, soft magnetic materials, hysteresis modeling

## Abstract

This paper presents investigation results regarding the Jiles-Atherton-based hysteresis loop modeling of ultra-high permeability amorphous alloy MELTA^®^ MM-5Co. The measurement stand is capable of accurately measuring minor and major hysteresis loops for such a material together with exemplary measurement results. The main source of the measurement error is highlighted, which includes the Earth’s field influence. The results of hysteresis loop modeling with the original Jiles-Atherton model and with two of its modifications are given. In all cases, the parameters of the Jiles-Atherton model were identified in two-step identification on the basis of a differential evolution optimization algorithm. The results indicate that both the original and modified Jiles-Atherton models are suitable for modeling the ultra-soft amorphous alloy. However, the hysteresis model’s parameters vary significantly.

## 1. Introduction

Demanding applications including aerospace technology require state-of-the-art materials such as ultra-high permeability alloys with quasistatic maximum relative permeability *µ* exceeding 250,000. Such materials are especially suitable for fluxgate sensors for precision measurements of magnetic fields [1,2,3]. It should be highlighted that, until now, over 200 fluxgate magnetometers were used in space research. During a past project concerned with the design of low-noise magnetic fluxgates, the modified MELTA^®^ MM-5Co amorphous alloy was chosen for the core of the device [4]. Reference [5] describes a low-noise (<1 pT) sensor based on similar amorphous material. Trying to apply this amorphous material in three-axis sensors was met with problems of excitation voltage unbalance. This could happen if the pieces of tape for each core are taken from different bobbins (even annealed in the same batch) or even from different parts of the same bobbin. For instance, from the inner, middle, and upper part of the bobbin. The following investigation is a follow-up of this project.

While it is common practice to construct such devices and investigate their functional characteristics later, the process of design optimization needs precise methods of computer modeling that allow us to find the best solution from a number of variables including geometric parameters of magnetic circuits. The correct model of the magnetic hysteresis loop is essential for optimization accuracy [6]. One such model is the Jiles-Atherton model (J-A model) [7], which already has a number of improvements and extensions including the temperature and stress dependence [8] of magnetic characteristics.

On the other hand, the Jiles-Atherton model is commonly considered a model suitable for modeling magnetic hysteresis loops of semi-hard materials [9]. The physical principles on which the model was based were the subject of criticism [10] with some critics even going as far as to describe it as unphysical. As a result, the modifications of the Jiles-Atherton model were proposed to better reflect the physical processes behind a magnetic hysteresis loop.

This paper fills the gap in state-of-the-art modeling of magnetic hysteresis loops of ultra-high permeability alloys. The parameters of the original Jiles-Atherton model and two of its most important variations were determined. The quality of modeling was assessed from the point of view of technical applications, i.e., for modeling the magnetic properties of ultra-high permeability alloys for magnetic cores of sensors for space applications.

## 2. Materials and Methods 

### 2.1. Ultrahigh Permeability Sample

The ring-shaped samples used in the investigation were made of amorphous alloy Co_67_Fe_3_Cr_3_B_12_Si_15_. It is a novel magnetic material developed as the modified version of the Co-based amorphous alloy MELTA^®^ MM-5Co [11] with a low saturation flux density *B_s_*, nearly zero magnetostriction *λ*, a Curie temperature of *T_C_* = 460 K, and remarkably soft magnetic properties. Magnetic cores made of Co_67_Fe_3_Cr_3_B_12_Si_15_ ribbons exhibit very high relative permeability. Its initial relative permeability *µ_i_* exceeds 180,000 and 135,000 for 1 and 10 kHz frequencies, respectively. Moreover, maximal relative permeability *µ_max_* exceeds 150,000 and 250,000 for these frequencies. It also exhibits low core losses of 0.2 and 4.42 W/kg for saturation at 1 and 10 kHz frequencies, respectively.

The magnetic properties of the Co_67_Fe_3_Cr_3_B_12_Si_15_ amorphous alloy can be improved by inducing magnetic anisotropy and by annealing in the magnetic field. After such processing, the hysteresis loop changes its shape and becomes squarer or flat depending on the direction and magnitude of the induced magnetic anisotropy.

**Figure 1:**
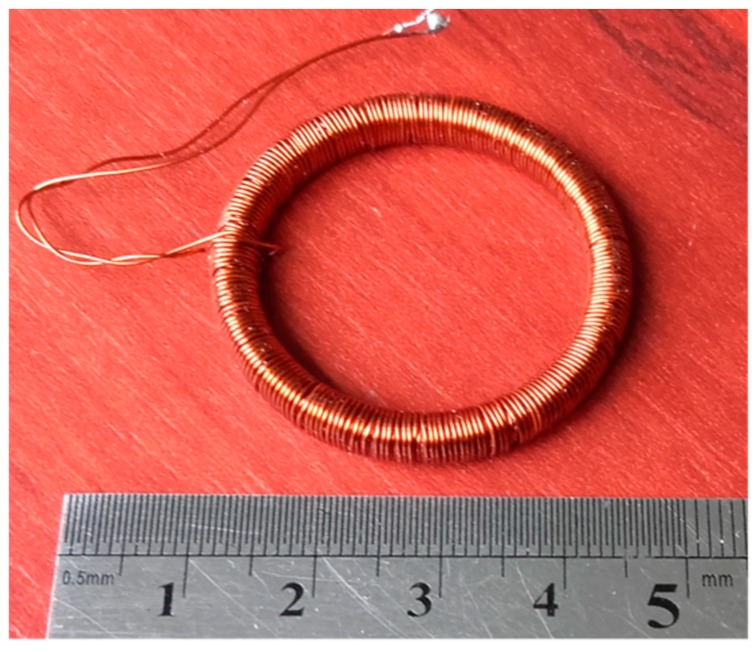
Typical sample used in the investigation, a ring-shaped core with a small cross-section, with 400 sensing windings, ensuring high measurement signal. Magnetization was provided with a straight magnetizing rod, which ensured a homogenous magnetizing field in the sample.

The material for samples was annealed for 1 h at 440 °C in a CO_2_ atmosphere. At the cooling stage (approximately at 250 °C), a transverse DC (Direct Current) field of 40 kA/m was applied until the core reached 100 °C. Then it was removed from the oven. The field was generated by a solenoid with a DC current. The material was in 25–30 mm bobbins, but only the small parts of the tape from the bobbins were rewrapped to 32 mm diameter supports (Figure 1).

**Figure 2:**
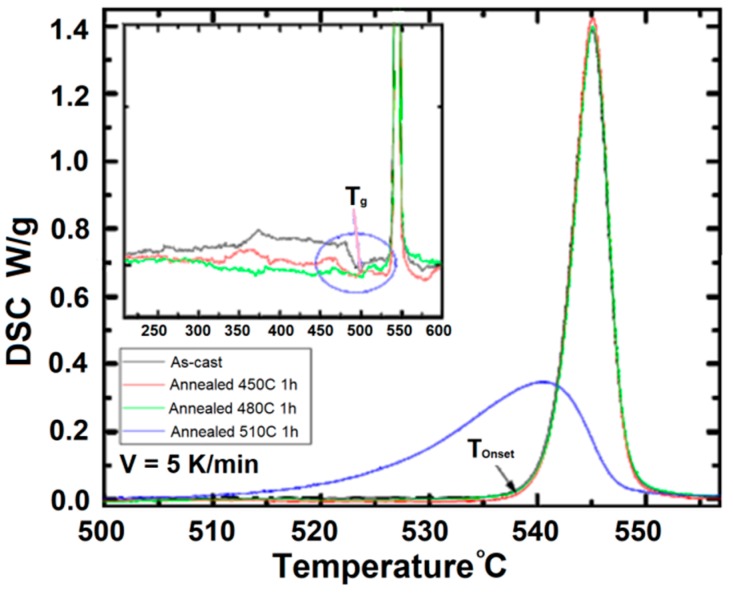
Differential scanning calorimetry investigation of the Co_67_Fe_3_Cr_3_B_12_Si_15_ amorphous alloy.

For an annealing temperature below 490 °C, the Co_67_Fe_3_Cr_3_B_12_Si_15_ cores are mainly amorphous. To investigate this, Differential Scanning Calorimetry (DSC 404 F1 Pegasus^®^, NETZSCH Group, Selb, Germany) tests were conducted and the results are presented in Figure 2. Each thermogram of differential scanning calorimetry has only a maximum, which is a sign of eutectic crystallization typical for this kind of alloy. A glass transition temperature T_g_ and the temperature of the onset of crystallization T_onset_ are shown by the arrows in Figure 2.

We can see in Figure 2 that the DSC curves of the as-cast ribbon (dark-grey line) and the ribbons annealed for 1 h at 450 °C (red line) and 480 °C (green line) are very similar. This means that the annealed tapes remained in an amorphous state. The DSC curve of the tape annealed for 1 h at 510 °C (blue line) is different from the other curves. The temperature of the onset of crystallization and the height of the maximum are lower than those of the other samples’ thermograms. These changes manifest the crystallite presence in the amorphous matrix.

The difference between the 440 °C annealing temperature of the samples and the temperature of the onset of crystallization T_onset_ is high enough to confirm the amorphous structure of the samples used in the following investigation. Moreover, the literature review hints at a slight increase of the crystallization temperature for amorphous ribbons annealed under the influence of a magnetic field [12,13,14,15,16]. The rate of the crystallization, however, was higher.

### 2.2. Measurement Method

**Figure 3:**
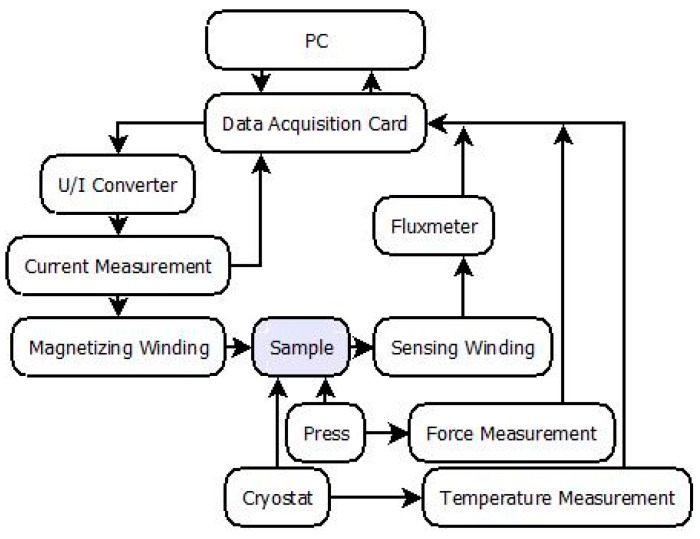
Schematic diagram of the developed Ferrograph system [17].

The measurements were carried out on the specially designed PC-controlled Ferrograph system (model Blacktower, ESP, Warsaw, Poland). Figure 3 presents the schematic diagram of this system. The most important part of the system is a PC equipped with an NI PCI-6221 DAQ card (National Instruments, Austin, TX, USA) connected to Lakeshore 480 fluxmeter (Lake Shore Cryotronics, Westerville, OH, USA) and a KEPCO BOP36-6M voltage-current converter (KEPCO, New York, NY, USA). The specialized software (Labview, v.11, National Instruments, Austin, TX, USA) controls the voltage-current converter, which supplies current to the magnetizing winding. The voltage induced in the measurement winding is connected to the input of the fluxmeter. The system is also equipped with a continuity tester to ensure that there is a separation between input and output connectors and continuity in the measurement winding (since this is the most common source of invalid measurements).

The Ferrograph was designed and constructed as a modular system currently equipped with temperature and stress-setting units, which allows for additional measurement flexibility (the system was described in reference [17]).

Due to the exceptional magnetic permeability of the samples and the high current output of the voltage-current converter (up to 6 A), typical magnetizing windings were replaced with a single axial magnetizing rod. As a result, the produced magnetizing field has better uniformity along the sample magnetic path than the magnetizing winding of the low winding count.

A magnetizing signal waveform can be chosen as sinusoidal, triangular, tangential, arbitrary, or automatically fit for a constant *dB*/*dt* in the given sample. In the presented research, the sample was magnetized by a triangular current signal with a frequency equal 0.1 Hz.

Additionally, three-axial Helmholtz coils were used to actively shield the measured sample from the influence of an external DC magnetic field.

## 3. Results

**Figure 4:**
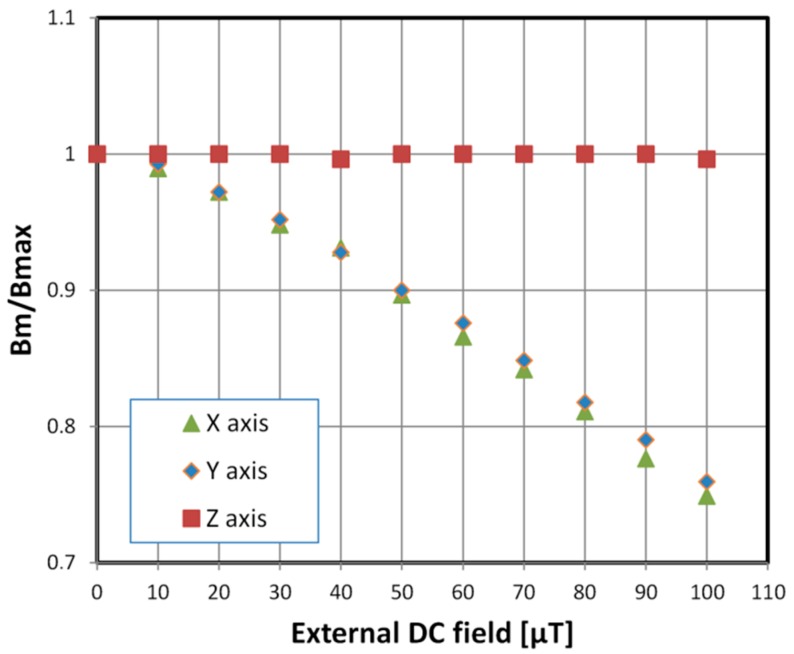
Dependence of measured maximal induction *B_m_* value on the external homogeneous field. Values normalized with a *B_max_* for an external field equal 0 ± 0.01 μT in all three axes. Each of the axes were investigated separately. The obtained values for fields in the plane of the ring sample (X and Y) are similar while the influence of the transverse field (Z axis, along the axis of the ring core) is negligible.

The investigated material exhibited exceptionally high permeability and low coerciveness while having the classical shape of the hysteresis loop. Because of the sample’s permeability, cancelling out the Earth’s field influence was essential for accurate measurements. Additionally, measurements of saturated hysteresis loops (with magnetizing field *H_m_* = 3 A/m) were taken under increasing external homogeneous magnetic field. Figure 4 presents the influence of this field on maximal induction *B_m_* for fields perpendicular to the sample axis (designated X and Y). It was found when taking measurements that omitting the Earth’s field (~50 μT) cancellation induces errors as high as 10%. Fields parallel to the sample axis (Z axis) had a negligible effect in the typical Earth field range.

**Figure 5:**
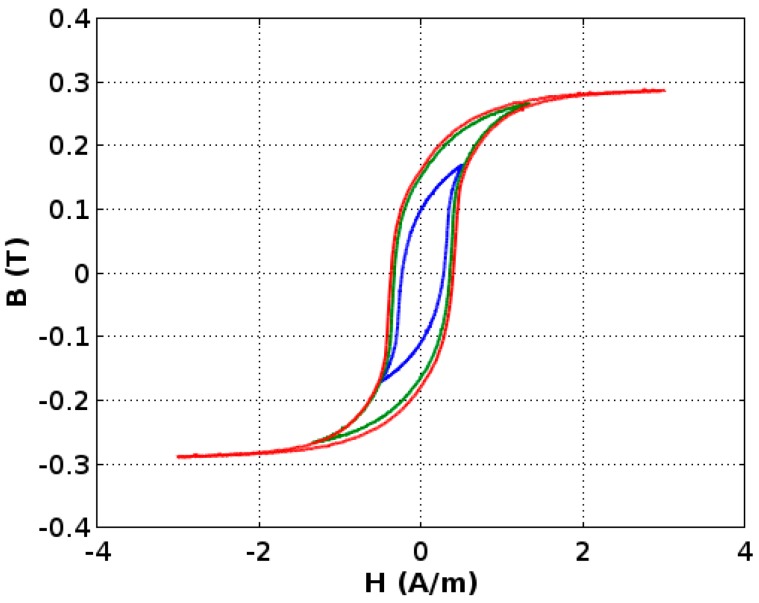
The measurement results for the ring-shaped core made of the amorphous Co_67_Fe_3_Cr_3_B_12_Si_15_ alloy was annealed for 1 h at 440 °C in the presence of magnetic field H, which is equal to 40 kA/m applied in the direction of the amorphous alloy ribbon. The maximal points of the minor hysteresis loops follow the normal magnetization curve (blue line—hysteresis loop for magnetizing field *H_m_* = 0.5 A/m; green line—*H_m_* = 1.5 A/m; red line—*H_m_* = 3 A/m).

Each measured loop consists of 10,000 measurement points. The system enables researchers to average an arbitrary number of results and enables additional filtration of the signal with a filter of a chosen order and a cut-off frequency. In the presented case, however (Figure 5), it was not necessary due to the low noise in the obtained results.

Each sample was measured in the same conditions and to check repeatability of the results. The spread of the hysteresis parameters averaged between the samples was smaller than 5%. The repeatability of the measurements for a single sample was ~1%. The extended uncertainty of the utilized Ferrograph system is 1%.

The following model was performed on results averaged from 50 consecutive measurements of one chosen sample (Figure 1).

### 3.1. Jiles-Atherton Hysteresis Model and Its Modifications

The Jiles-Atherton model of magnetic hysteresis is commonly used for physical and technological purposes due to its simplicity and possibility for the reproduction of magnetic hysteresis loops of both isotropic and anisotropic materials [18]. For this reason, the Jiles-Atherton model is widely implemented in SPICE software for modeling magnetic circuits in electronics components such as in references [19,20,21].

The Jiles-Atherton model is based on the anhysteretic magnetization curve calculated on the base of the Bozorth distribution. For uniaxial anisotropy, the anhysteretic magnetization curve is given by the following equation [22,23].

(1) $$M_{ah}=M_{s}\frac{\int_{0}^{\pi }e^{\frac{E\left(1\right)+E\left(2\right)}{2}}sin\theta \cdot cos\theta \;d\theta }{\int_{0}^{\pi }e^{\frac{E\left(1\right)+E\left(2\right)}{2}}sin\theta \;d\theta }$$

where:

(2) $$E\left(i\right)=\frac{H_{e}}{a}\cos\theta -\frac{K_{an}}{M_{s}\mu_{0}a}\sin^{2}\phi_{i}$$

In the above equations, *M_s_* is the saturation magnetization of magnetic materials, *K_an_* is the average uniaxial anisotropy energy density, *a* quantifies the domain wall density, *φ*_1_ = (*ψ* − *θ*), and *φ*_2_ = (*ψ* + *θ*) where *ψ* is the angle between the magnetization direction and the easy axis of magnetized material. According to the Bloch model, an effective magnetizing field is defined by *H_e_* = *H* + *αM* where *α* Bloch’s is inter-domain coupling and *M* is the total magnetization of the sample. For the isotropic materials where *K_an_* = 0, Equation (1) reduces to the Langevin function below.

(3) $$M_{ah}=M_{s}\left[coth\left(\frac{H_{e}}{a}\right)-\frac{a}{H_{e}}\right]$$

Magnetic hysteresis in the Jiles-Atherton model is introduced by the ordinary differential equation determining *dM*/*dH*. In the original Jiles-Atherton model, on the basis of reference [7], the equation below is found.

(4) $$\int \mu_{0}M_{ah}\left(H\right)dH_{e}=\int \mu_{0}M\left(H\right)dH_{e}+\delta k\mu_{0}\int dM\left(H\right)$$

The following differential equation is stated.

(5) $$\frac{dM}{dH}=\frac{\delta_{M}}{\left(1+c\right)}\frac{\left(M_{an}-M\right)}{\left(\delta k-\alpha \left(M_{an}-M\right)\right)}+\frac{c}{\left(1+c\right)}\frac{dM_{an}}{dH}$$

However, considering the assumption given in reference [7], the equation below is determined.

(6) $$\int \mu_{0}M_{ah}\left(H\right)dH_{e}=\int \mu_{0}M\left(H\right)dH_{e}+\delta k\mu_{0}\int dM_{irr}\left(H\right),$$

the alternative form of a differential equation for magnetic hysteresis was presented by P. Cheng et al. [23] determined the formula below.

(7) $$\frac{dM}{dH}=\frac{\delta_{M}\left(1-c\right)\left(M_{an}-M\right)+c\delta k\frac{dM_{an}}{dH}}{\delta k-\alpha \left(1-c\right)\left(M_{an}-M\right)}$$

Alternatively, R. Venkataraman et al., considering physical analysis-based dependences [24] indicated that:

(8) $$\int \mu_{0}M_{ah}\left(H\right)dH_{e}=\int \mu_{0}M\left(H\right)dH_{e}+\delta k\mu_{0}\left(1-c\right)\int dM_{irr}\left(H\right)$$

which leads to another form of the Jiles-Atherton model-based equation.

(9) $$\frac{dM}{dH}=\frac{\frac{k\delta }{\mu_{0}}c\frac{dM_{ah}}{dH_{e}}+\delta_{M}\left(M_{ah}-M\right)}{\frac{k\delta }{\mu_{0}}-\delta_{M}\left(M_{ah}-M\right)\alpha -\frac{k\delta }{\mu_{0}}\alpha c\frac{dM_{ah}}{dH_{e}}}$$

It should be stressed that all proposed approaches to hysteresis given by Equations (4)–(9) consider the same set of parameters: *M_irr_*, irreversible magnetization, *c*, reversibility of the magnetization process, *k*, quantifies the average energy required to the break pining site, *δ*, determines if magnetizing the field increases or decreases while *δ_M_* is necessary for the avoidance of unphysical stages of the Jiles-Atherton model for minor loops where incremental susceptibility becomes negative [19].

It should be highlighted that there are severe computational problems connected with the Jiles-Atherton model of magnetic hysteresis. These include the method of numerical integration (for the anisotropic anhysteretic curve stated in Equation (1) as well as solving differential equations given by Dependences (5), (7), and (9). Numerical integration may be carried out by using trapezoidal elements methods or the Gaus–Kronrod interpolation. However, for solving differential equations, the Runge-Kutta method ODE solver has to be applied [25]. The Riemann method may lead to significant differences due to the accumulation of numerical errors during the integration process. 

Additionally, there is no direct method available for the calculation of the Jiles-Atherton model’s parameters on the basis of measured hysteresis loops. Instead, identification of the model’s parameters can be performed on the basis of optimization algorithms such as the differential evolution [26]. It allows omits local minima of the target function. A two-step identification method of the Jiles-Atherton model parameters was proposed previously [27].

**Step 1**: calculate anhysteretic parameters: *M_s_*, *a*, *α*, and *K_an_*.

**Step 2**: introduce hysteresis and find hysteresis parameters *k* and *c*.

Target function *F* for the optimization process was given by the following equation.

(10) $$F=\sum_{i=1}^{n}{\left(B_{meas}\left(H_{i}\right)-B_{sym}\left(H_{i}\right)\right)}^{2}$$

Lastly, all parameters are adjusted during the normalized Nelder–Mead simplex optimization algorithm.

### 3.2. Modeling Results

**Figure 6:**
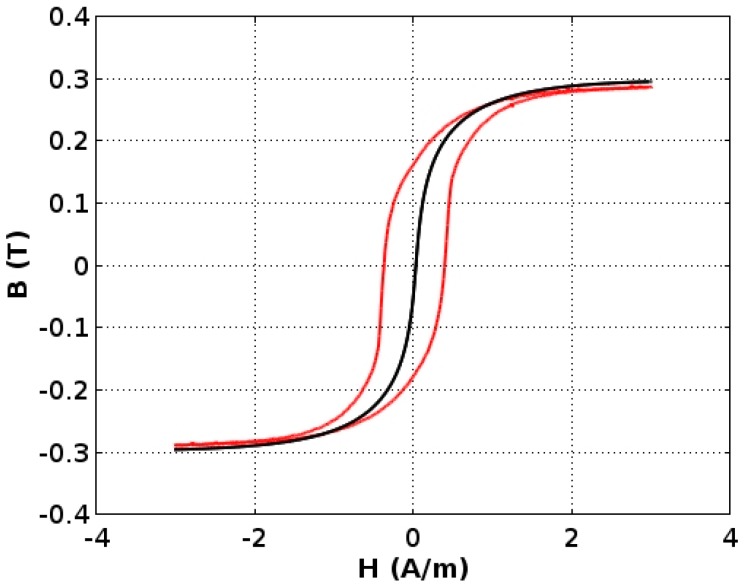
An anhysteretic magnetization curve (black line) is determined in Step 1 on the base of major hysteresis loop measurement results (red line).

As was indicated in Section 3, parameters of the anhysteretic curve were identified in the first step. As known from the production process, a uniaxial anisotropy easy axis of a ring-shaped core was generated in the direction of the ribbon. As a result, parameter *ψ* = 0. Figure 6 presents the results of identification of the anhysteretic curve, which is the same for all three versions of the Jiles-Atheron model.

In the second step, parameters *c* and *k* of the Jiles-Atherton-based models were simultaneously determined for three hysteresis loops measured for different amplitudes of the magnetizing field. The shape of the hysteresis loop for the model given by Equation (7) is presented in Figure 6 while parameters of the models are presented in Table 1.

**Figure 7:**
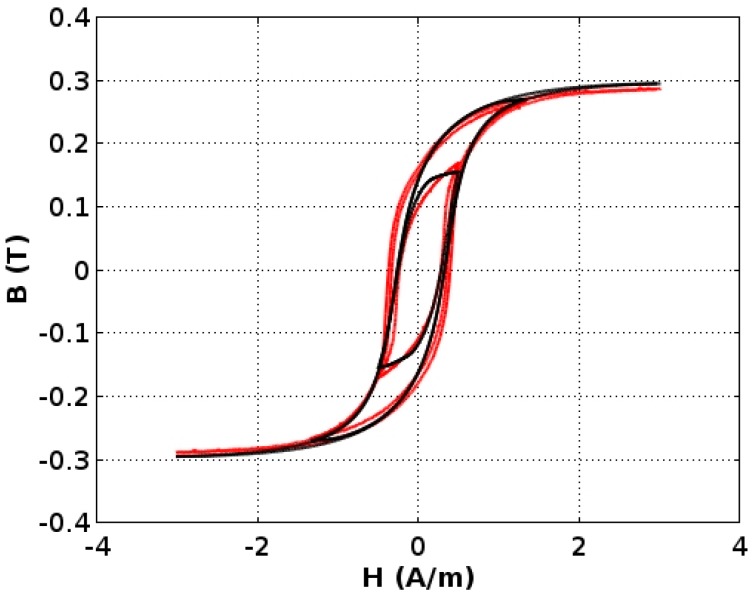
Modeling results (black lines) according to Equation (7). The measurement results are presented as red lines.

The presented results indicate that all three models represent the results of measurements of the magnetic hysteresis loops quite well. The P. Cheng et al. model given by Equation (7) exhibits the best agreement with the experimental results. This agreement is confirmed by the high value of the determination coefficient R^2^, which exceeds 0.992. This indicates that the P. Cheng et al. version of the Jiles-Atherton model is the most suitable for modeling the magnetic characteristics of the investigated ultra-high permeability alloy for space applications. Modeled hysteresis loops compared with the measurement results are presented in Figure 7. Modeling accuracy is better than the extended uncertainty of the measurement. Therefore, it is treated as negligible.

## 4. Conclusions

The measurement stand capable of hysteresis measurements of ultra-high permeability samples was presented. The paramount importance of stray homogeneous fields shielding during measurements was highlighted. Exemplary measurement results, together with the characteristics of the Earth’s field influence on MELTA^®^ MM-5Co, were given. The obtained measurement data were used for the Jiles-Atherton model parameter identification in its basic and modified forms. The presented results confirm that the Jiles-Atherton model is suitable for modeling the magnetic characteristics of Co_67_Fe_3_Cr_3_B_12_Si_15_ ultra-high permeability magnetic material. Moreover, for modeling the Co_67_Fe_3_Cr_3_B_12_Si_15_ amorphous alloy for space applications, the most adequate results may be achieved by utilizing the J-A model modification proposed by P. Cheng et al. High accuracy of the model given by Equation (7) is confirmed by the determination coefficient R^2^, which exceeds 0.992. The reason may be due to the fact that the P. Cheng et al. version is believed to better reflect the physical processes behind ferromagnetic hysteresis. 

The obtained results are currently used in the follow-up of the “Small Explorer for Advanced Missions” [4] and “Digital Magnetometer for Microsatellites Lemi-020” [28] projects, which are focused on the design and development of magnetic fluxgate sensors for space applications with a main goal of lowering noise. 

## Figures and Tables

**Table 1:** Parameter identification results for the three models.

Parameter	Unit	Jiles-Atherton	Venkataraman et al.	Cheng et al.
*M_s_*	A/m	219,600	216,600	237,200
*a*	A/m	0.269	0.268	1.669
*α*	-	2.824 × 10^−8^	1.151 × 10^−8^	6.268 × 10^−6^
*K_an_*	J/m^3^	2.383	32.08	1230
*ψ*	rad	0	0	0
*k*	A/m	0.318	0.322	0.263
*c*	-	0.0001	0.000	0.459
R^2^	-	0.984	0.984	0.992

## References

[1] Ripka P. (1992). Review of fluxgate sensors. Sens. Actuators A Phys..

[2] Primdahl F. (1979). The fluxgate magnetometer. J. Phys. E Sci. Instrum..

[3] Forslund A., Belyayev S., Ivchenko N., Olsson G., Edberg T., Marusenkov A. (2008). Miniaturized digital fluxgate magnetometer for small spacecraft applications. Meas. Sci. Technol..

[4] Small Explorer for Advanced Missions. http://www.isr.lviv.ua/SEAM.htm.

[5] Marusenkov A. (2017). Possibilities of further improvement of 1s fluxgate variometers. Geosci. Instrum. Methods Data Syst..

[6] Ando B., Baglio S., La Malfa S., Bulsara A.R. (2012). Adaptive Modeling of Hysteretic Magnetometers. IEEE Trans. Instrum. Meas..

[7] Jiles D.C., Atherton D.L. (1986). Theory of ferromagnetic hysteresis. J. Magn. Magn. Mater..

[8] Jackiewicz D., Szewczyk R., Salach J. (2013). Mathematical and computer modelling of the influence of stress on magnetic characteristics of the steels. Theor. Appl. Inform..

[9] Pop N.C., Caltun O.F. (2011). Jiles-Atherton magnetic hysteresis parameters identification. Acta Phys. Pol. A.

[10] Zirka S.E., Moroz Y.I., Harrison R.G., Chwastek K. (2012). On physical aspects of the Jiles-Atherton hysteresis models. J. Appl. Phys..

[11] Nosenko V.K., Maslov V.V., Kirilchuk V.V., Kochkubey A.P. (2008). Some industrial applications of amorphous and nanocrystalline alloys. J. Phys. Conf. Ser..

[12] Onodera R., Kimura S., Watanabe K., Yokoyama Y., Makino A., Koyama K. (2013). Magnetic field effects on crystallization of iron-based amorphous alloys. Mater. Trans..

[13] Wolfus Y., Yeshurun Y., Felner I., Wolny J. (1987). Crystallization kinetics in amorphous ferromagnets effect of temperature and magnetic field. Philos. Mag. B.

[14] Yu Y., Liu B., Qi M. (2008). Crystallization behavior of Fe_78_Si_13_B_9_ metallic glass under high magnetic field. J. Univ. Sci. Technol. Beijing.

[15] Rivoirard S. (2013). High steady magnetic field processing of functional magnetic materials. JOM.

[16] Milyutin V.A., Gervaseva I.V., Beaugnon E., Gaviko V.S., Volkova E.G. (2017). The process of crystallization from amorphous state in ribbons of Fe–Si–B–based alloys under the effect of a high DC magnetic field. Phys. Met. Metallogr..

[17] Charubin T., Nowak P.T., Nowicki M., Szewczyk R., Urbański M. (2018). Automatic measurement station for ferrite materials testing. Acta Phys. Pol. A.

[18] Ramesh A., Jiles D.C., Roderik J. (1999). A model of anisotropic anhysteretic magnetization. IEEE Trans. Magn..

[19] Ando B., Baglio S., La Malfa S., Bulsara A.R. (2011). SPICE simulation of coupled core fluxgate magnetometers. Proceedings of the 2011 IEEE International Instrumentation and Measurement Technology Conference.

[20] Ando B., Baglio S., Bulsara B., La Malfa S. (2010). RTD Fluxgate behavioral model for circuit simulation. Procedia Eng..

[21] Gaskill S.G., Weisshaar A. (2016). Compact equivalent circuit modeling of microfluxgate devices with thin-film magnetic cores. IEEE Trans. Magn..

[22] Szewczyk R. (2014). Validation of the anhysteretic magnetization model for soft magnetic materials with perpendicular anisotropy. Materials.

[23] Cheng P., Szewczyk R. (2018). Modified description of magnetic hysteresis in Jiles-Atherton model. Adv. Intell. Syst. Comput..

[24] Venkataraman R., Krisnaprasad P.S. (1998). Qualitative analyse of a bulk ferromagnetic hysteresis model. Proceedings of the 37th IEEE Conference on Decision and Control.

[25] Szewczyk R. (2014). Computational problems connected with Jiles-Atherton model of magnetic hysteresis. Adv. Intell. Syst. Comput..

[26] Biedrzycki R., Jackiewicz D., Szewczyk R. (2014). Reliability and efficiency of differential evolution based method of determination of Jiles-Atherton model parameters for X30Cr13 corrosion resisting martensitic steel. J. Autom. Mob. Robot. Intell. Syst..

[27] Szewczyk R. (2018). Two step, differential evolution-based identification of parameters of Jiles-Atherton model of magnetic hysteresis loops. Adv. Intell. Syst. Comput..

[28] Digital Magnetometer for Microsatellites LEMI-020. http://www.isr.lviv.ua/lemi020.htm.

